# Surface tension measurement and calculation of model biomolecular condensates

**DOI:** 10.1039/d3sm00820g

**Published:** 2023-09-20

**Authors:** Jack Holland, Alfonso A. Castrejón-Pita, Remco Tuinier, Dirk G. A. L. Aarts, Timothy J. Nott

**Affiliations:** a Department of Chemistry, Physical and Theoretical Chemistry Laboratory, University of Oxford Oxford OX1 3QZ UK dirk.aarts@chem.ox.ac.uk; b Dept. of Biochemistry, University of Oxford Oxford OX1 3QU UK tim.nott@bioch.ox.ac.uk; c Dept. of Engineering Science, University of Oxford Oxford OX1 3PJ UK; d Laboratory of Physical Chemistry, Department of Chemical Engineering and Chemistry & Institute for Complex Molecular Systems (ICMS), Eindhoven University of Technology 5600 MB Eindhoven The Netherlands

## Abstract

The surface tension of liquid-like protein-rich biomolecular condensates is an emerging physical principle governing the mesoscopic interior organisation of biological cells. In this study, we present a method to evaluate the surface tension of model biomolecular condensates, through straighforward sessile drop measurements of capillary lengths and condensate densities. Our approach bypasses the need for characterizing condensate viscosities, which was required in previously reported techniques. We demonstrate this method using model condensates comprising two mutants of the intrinsically disordered protein Ddx4^N^. Notably, we uncover a detrimental impact of increased protein net charge on the surface tension of Ddx4^N^ condensates. Furthermore, we explore the application of Scheutjens–Fleer theory, calculating condensate surface tensions through a self-consistent mean-field framework using Flory–Huggins interaction parameters. This relatively simple theory provides semi-quantitative accuracy in predicting Ddx4^N^ condensate surface tensions and enables the evaluation of molecular organisation at condensate surfaces. Our findings shed light on the molecular details of fluid–fluid interfaces in biomolecular condensates.

## Abstract 2

Straightforward sessile drop measurements of surface tension for model biomolecular condensates comprising phase-separated Ddx4^N^ are presented. We compare these with theoretical calculations, based around the self-consistent Scheutjens–Fleer theory.

## Graphical abstract

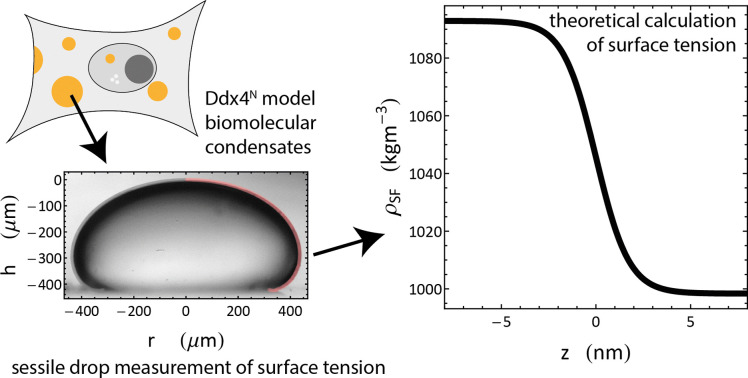

## 1 Introduction

Many physiological processes necessary to sustain life are associated with liquid-like membraneless intracellular compartments or ‘biomolecular condensates’.^1^ Interfacial phenomena,^2^ driven by surface tension, facilitate their intracellular localisation through wetting,^3–8^ regulated coalescence^9^ and the formation of multiphase condensates.^10^ Understanding how interactions between chemical components of biomolecular condensates contribute to their surface tension is, thus, necessary for molecular interpretations of such phenomena. Biomolecular condensates are often stabilised by weak multivalent interactions between intrinsically disordered proteins, whose sequences drive self-assembly through liquid–liquid phase-separation,^11^ generating protein-rich condensates surrounded by a protein-poor aqueous phase. Here, we seek to advance the physical understanding of biomolecular condensate surface tension by studying the fluid–fluid interface of model condensates formed by the intrinsically disordered protein (IDP) Ddx4^N^, using both experimental and theoretical approaches.

Rich in glycine, serine, aromatic and charged amino acids, Ddx4^N^ shares essential sequence features of many well-characterised condensate proteins such as FUS, hnRNPA1, PGL-3 and LAF-1,^12–15^ and is an established model biomolecular condensate system.^16–20^ Such model systems offer a degree of control over experimental parameters not currently feasible in living cells, where native biomolecular condensates comprise tens to hundreds of unique molecular species.^21^ Furthermore, physical principles governing the stability of model biomolecular condensates, prepared in aqueous buffer, are often mirrored by intracellular condensates. For example, we,^16,19^ and others,^13^ have shown favourable electrostatic interactions between charged amino acids of IDPs to stabilise condensates in both idealised buffer conditions, and living cells.

The surface tension of several model biomolecular condensates has been recorded, using techniques such as micropipette aspiration,^22^ capillary wave analysis,^23^ measurement of their coalescence rates^10,15,23–26^ and characterisation of their deformation by gravity.^10,27^ Reported values lie in the range 0.1–100 μN m^−1^,^10,22^ with physical interpretations leaning on scaling arguments, roughly characterising the energy density at the interface as the ratio of thermal energy to the square of a characteristic molecular dimension.^10,15,27^ Jawerth *et al.*^23^ showed the surface tension of PGL-3 condensates (1–5 μN m^−1^) decreases with increasing salt concentration according to the Overbeek–Voorn model, analogous to behaviour observed for complex coacervates formed by polyelectrolytes.^28^ Their result emphasises the role of electrostatic interactions between protein chains at the surfaces of condensates in contributing to the observed surface tension. Otherwise, there is hitherto little physical understanding of the relationship between protein–protein interactions and the surface tension of their biomolecular condensates. Building on our previous investigations showing the detrimental effect of increased absolute protein net charge on Ddx4^N^ phase-separation,^19^ we seek to uncover the influence of Ddx4^N^ net charge on it's condensate's surface tension.

The phase behaviour of Ddx4^N^ and other phase-separating IDPs, PGL-3^29^ and hnRNPA1,^30^ can be described by Flory–Huggins solution theory,^16,18,29,30^ predicting phase diagrams by absorbing all intermolecular interactions into a single mean-field interaction parameter whilst assuming random mixing of chemical components. Flory–Huggins theory alone is unsuitable for modelling the interfacial region separating two phases, which is defined by non-random gradients in solution composition across the interface. We have previously modelled the surface tension of a demixed colloid-polymer solution, of magnitude 1 μN m^−1^, by augmenting a mean-field free energy expression with a van der Waals term accounting for gradients in composition.^31,32^ Scheutjens and Fleer notably developed a similar procedure,^33,34^ extending the Flory–Huggins theory for polymer phase-separation,^35,36^ accounting for gradients in composition using a self-consistent numerical lattice model. This approach has been applied to a variety of liquid–liquid interfaces, facilitating calculation of surface tensions and compositional profiles normal to the interface, revealing the manifest arrangement of molecules associated with surface tension.^37–40^ Despite its utility, Scheutjens–Fleer theory has not yet been applied to the surfaces of biomolecular condensates.

Here, we measure the surface tension of Ddx4^N^ model condensates and their response to the manipulation of protein net charge, and investigate the modelling capability of Scheutjens–Fleer theory to the same interfaces. We work with two Ddx4^N^ proteins, Ddx4^N^ 1-229 and Ddx4^N^ 1-231, differing in length by two amino acids and by a unit charge in 150 mM NaCl, 20 mM Tris pH 8.0. Ddx4^N^ 1-229 is related to Ddx4^N^ 1-231, through a two residue truncation at its C-terminus, with Ddx4^N^ 1-229 lacking the terminal EA dipeptide. Modulating the net charge in this way minimizes disruption of the characteristic patterning of alternating tracts of acidic and basic residues along the Ddx4^N^ chain (see Fig. 4(c)), known to enhance favourable electrostatic interactions driving its phase separation.^16,41^ See Sections 6.2, 6.3 and 6.4 for the sequences of the Ddx4^N^ proteins, details of their purification and their characterisation protocols.

In Section 2.1 we present a straightforward procedure, inspired by the work of Ijavi *et al.*,^27^ to determine surface tension from capillary lengths and condensate densities from bright-field images of sessile condensates. Fundamental aspects of Scheutjens–Fleer theory are given in Section 2.2, with emphasis on its relationship to Flory–Huggins theory. The results of our experiments and theoretical calculations are given in Section 3, which also includes a Scheutjens–Fleer characterisation of the molecular arrangements of Ddx4^N^ in the interfacial region. In Section 4 the wider implications of our results are discussed, with concluding remarks given in Section 5.

## 2 Theoretical background

### 2.1 Sessile drop measurement of interfacial tension

Earth's gravitational field deforms sessile droplets resting on flat substrates, pushing them towards a puddle-like geometry. This deformation is accompanied by an increase in droplet surface area, thus is counterbalanced by the droplet's surface tension. Balancing gravitational and surface forces gives rise to a characteristic length scale, the capillary length, defined as1
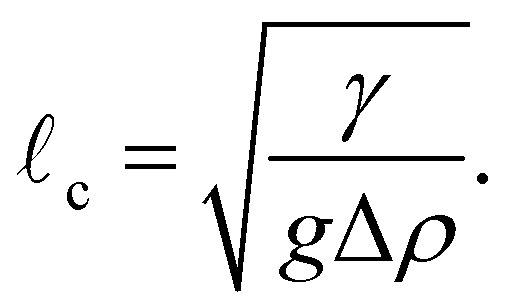
Here, *g* is gravitational acceleration (taken to be 9.81 m s^−2^), Δ*ρ* is the density difference between the droplet and its surrounding fluid phase and *γ* is the surface tension at the fluid–fluid interface of the drop. Droplets with characteristic length greater than ℓ_c_ exhibit gravitational deformation, whilst the geometry of those smaller than ℓ_c_ are dominated by capillary forces.^42^

**Fig. 1:**
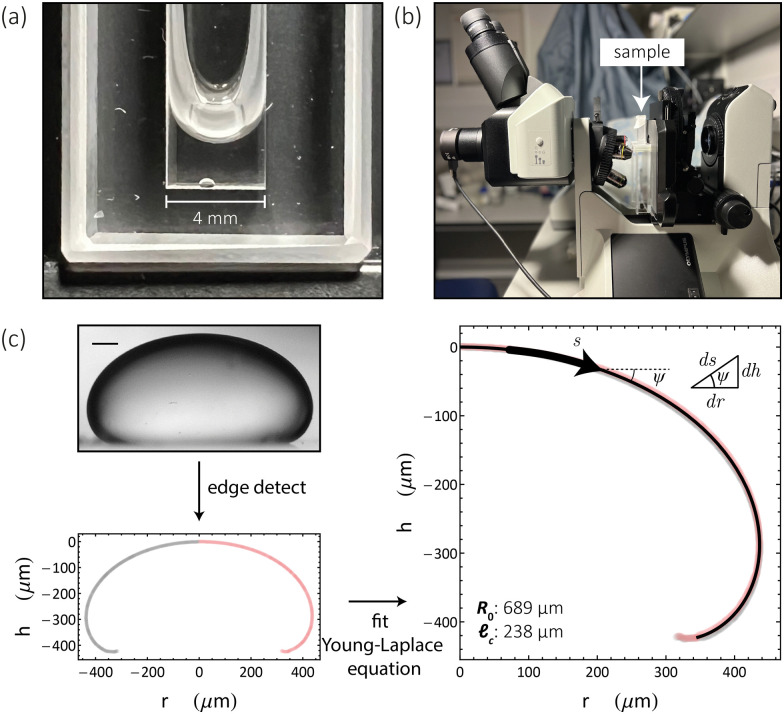
(a) Image showing a sessile condensate formed through centrifugation of 120 μL phase-separated Ddx4^N^ 1-231 in a PEGylated quartz glass cuvette (see Section 6.6). (b) Image of our microscopy set up. Images in (a) and (b) were captured using an iPhone 13 pro. (c) The fluid–fluid interface in bright-field images of sessile condensates (Ddx4^N^ 1-231 condensate shown) are identified as edges (scale bar 100 μm). Fitting of the Young-Laplace equation (eqn (2)) to imaged interfaces allows measurement of condensate capillary length and volume for subsequent determination of surface tension. Inset are diagrammatic definitions of the variables and specific values of the fitting parameters in eqn (2). The volume of this condensate was measured as 0.19 μL in a total solution volume of 80 μL (*ν*_β_/*ν*_0_ = 2.38 × 10^−3^).

A sessile drop exhibiting full rotational symmetry about its *h* axis, parallel to a uniform gravitational field, has the following Young–Laplace equation describing its fluid–fluid interface^43–47^2
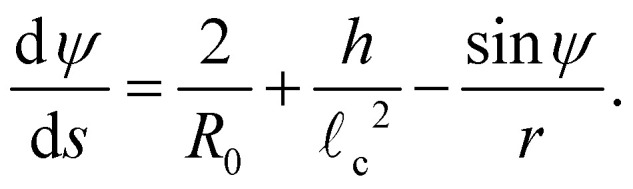
Here *s* is the distance along the interface from the apex, *ψ* is its angle incidence relative to the horizontal, *R*_0_ is the curvature at the apex and *r* is the radius at height *h*, with the origin of coordinates situated at the apex of the drop. Integration of eqn (2) over *s* generates theoretical interfaces, defined by *R*_0_ and ℓ_c_, which can be compared with images of sessile drops (see Fig. 1), collected as detailed in Section 6.6, enabling a measure of the capillary length. The fitting of eqn (2) to a sessile Ddx4^N^ condensate is illustrated in Fig. 1(c). If the density difference Δ*ρ* is known, then the surface tension *γ* can be determined from the capillary length through eqn (1). This is a standard technique used to determine the interfacial tension of macroscopic liquid droplets^48^ of known density, and more recently, the interfacial tension of microscopic biomolecular condensates,^27^ whose density was determined from sedimentation velocity measurements.

We determine the density difference Δ*ρ* = *ρ*_β_ − *ρ*_α_, by considering mass and volume conservation upon the formation of protein-rich phase β and protein-poor phase α, through phase-separation, to give3
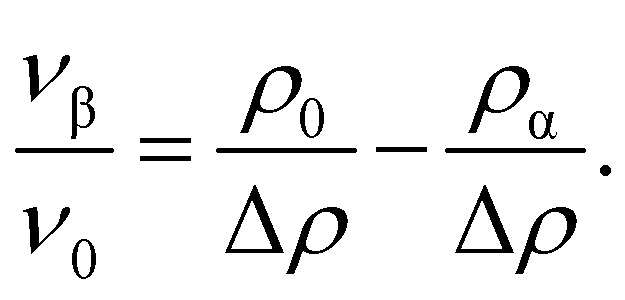
The total volume of the phase-separated solution and its density are represented by *ν*_0_ and *ρ*_0_. *ρ*_α_ and *ν*_β_ denote the density of the protein-poor phase and total volume of the condensed protein phase respectively. In our system, Ddx4^N^ phase-separation is initiated as a sample is cooled through its transition temperature, nucleating numerous microscopic condensates (see Section 6.6). Inducing the coalescence of these Ddx4^N^ microdroplets through centrifugation gives a single large condensate, examples of which are shown in Fig. 1(a) and (c), imaged using an iPhone 13 pro rear camera and bright-field microscopy respectively. The volume *ν*_β_, of the condensate is readily obtainable by fitting eqn (2) to our cross-sectional images captured through bright-field microscopy (see Fig. 1(b) and (c) and Sections 6.6 and 6.7). If our phase-separated solution behaves as a binary mixture of solvent and protein, as we have observed previously,^16,18^Eqn (3) predicts a linear relationship between *ν*_β_/*ν*_0_ and *ρ*_0_, when *ρ*_0_ is varied by altering protein concentration only (see Section 6.5). The gradient of this linear relationship yields 1/Δ*ρ*, thus Δ*ρ* may be determined by plotting *ν*_β_/*ν*_0_ against *ρ*_0_ and performing linear regression. Furthermore, the vertical-intercept is dependent on *ρ*_α_, which we are able to determine through spectrophotometry (see Sections 6.4 and 6.5), allowing further verification of both eqn (3) and the binary mixture approximation. With knowledge of the density difference across the interface and the capillary length, condensate surface tension is evaluated through eqn (1).

### 2.2 Scheutjens–Fleer calculation of interfacial tension

The bulk phase behaviour of phase-separating proteins, such as Ddx4^N^, may be described on a mean-field level by Flory–Huggins theory.^16,35,36^ This models a protein solution as homopolymer chains and solvent distributed on a lattice, where each site is occupied by either a monomer or solvent. For a solution comprising *ϕ*_p_ volume fraction of freely jointed protein chains, comprising *N*_p_ monomers, the Flory–Huggins mixing free energy per lattice site is4
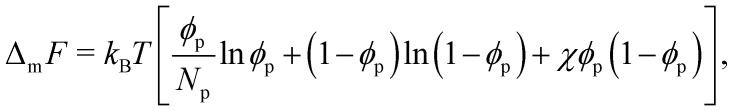
where the logarithmic terms give the configurational entropy of the lattice, and the terms in *χ* characterize the mean-field interaction between monomer and solvent sites. Scheutjens and Fleer have extended eqn (4) to account for the gradients in composition present in interfacial regions, allowing the evaluation of surface tensions.^33,34^ The essential features of Scheutjens–Fleer theory are now outlined, for more details see Section 6.8 and references therein.

For a binary lattice of polymer and solvent with gradient in *ϕ*_p_ only along the *z*-axis, the mixing free energy can be written as5
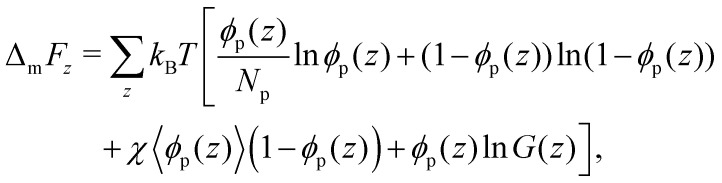
where the sum is over sites along the *z* axis. The penultimate term gives the mixing energy, with angular brackets denoting a weighted average over adjacent *z* layers;

6 〈*ϕ*_p_(*z*)〉 = *λϕ*_p_(*z* − 1) + (1 − 2*λ*)*ϕ*_p_(*z*) + *λϕ*_p_(*z* + 1).

Here, *λ* is the proportion of adjacent lattice sites in the (*z* + 1)th or (*z* − 1)th layers, which we set to 1/6 representing a cubic lattice. The final term in eqn (5) gives the extra mixing entropy associated with the interfacial region,^33^ with7
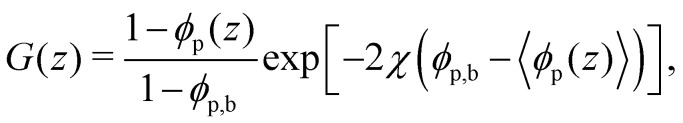
where *ϕ*_p,b_ denotes the volume fraction of polymer in one of the coexisting bulk phases.

The equilibrium lattice configuration is given by the compositional profile *ϕ*_p_(*z*) which minimizes eqn (5). Within the Scheutjens–Fleer framework, this is found by considering an initial profile *ϕ*_p_(*z*), from which potentials *u*(*z*) are calculated for each *z* slice using the Flory–Huggins field.^49,50^ The configuration is relaxed according to the Edwards diffusion equation, ensuring the placement of freely-jointed polymer segments adheres to the Boltzmann distribution.^37,51^ Iteration of this process until convergence of *ϕ*_p_(*z*) yields the optimum interfacial profile, from which the surface tension is obtained by calculating the grand potential^52^8
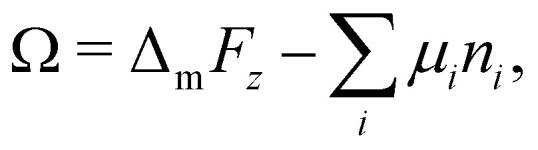
which in our system, is equal to the interfacial energy *γb*^2^. Here *b* is the lattice size in metres, *μ*_*i*_ is the chemical potential of species *i* and *n*_*i*_ is the number of species *i*. The lattice size sets the distance between adjacent lattice sites, which we define as the cube root of the mean molecular volume per amino acid for each Ddx4^N^ protein (calculated as detailed in Section 2.1), amounts to 5.12 Å (to 3 s.f.) for both Ddx4^N^ proteins.

For lattice sites in either coexisting equilibrium bulk phase, away from the interface, eqn (5) simplifies to eqn (4). According to Flory–Huggins theory, the binodal curve relating the compositions of coexisting phases to the interaction parameter is given by^18^9
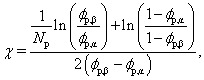
where *ϕ*_p,β_, and *ϕ*_p,α_, are the coexisting volume fractions of protein inside and outside condensates respectively, which we calculate using our experimentally measured densities *ρ*_α_ and Δ*ρ* (see Section 6.5). Application of eqn (9) therefore permits the extraction of a mean-field interaction parameter describing the phase-separation of each of our Ddx4^N^ condensates, affording a comparison with theoretical Scheutjens–Fleer calculations of surface tension. We note that eqn (9) does not consider the effects of droplet curvature on the phase equilibria. In Section 7.1 we show that, for the typical length and energy scales in our system, we expect the impact of curvature effects on the coexisting volume fractions to be of order 10^−3^%.

## 3 Results

**Fig. 2:**
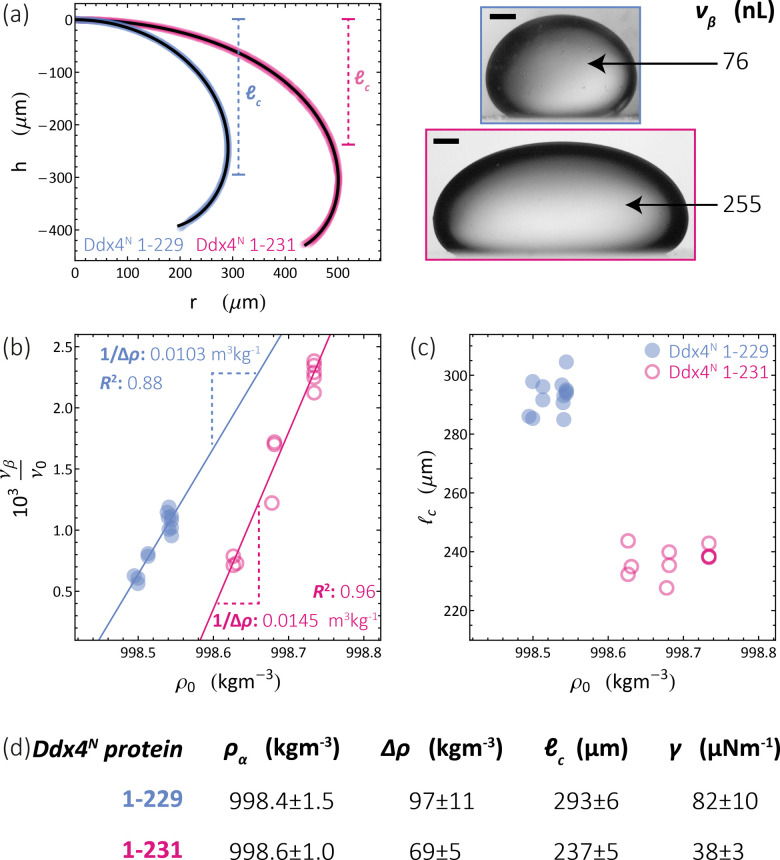
(a) Young–Laplace fits to sessile Ddx4^N^ 1-229 and Ddx4^N^ 1-231 condensates adjacent to the corresponding bright-field images (scale bars 100 μm). (b) Measured values of the ratio of condensate to sample volume *ν*_β_/*ν*_0_ plotted against sample densities *ρ*_0_ for Ddx4^N^ 1-229 (blue) and Ddx4^N^ 1-231 (magenta). Solid lines indicate linear fits, with gradients related to 1/Δ*ρ* through eqn (3). (c) Measured capillary lengths ℓ_c_ plotted against sample densities *ρ*_0_ for Ddx4^N^ 1-229 (blue) and Ddx4^N^ 1-231 condensates (magenta). (d) Table displaying parameters obtained from sessile drop analysis. Uncertainties in *ρ*_α_ and Δ*ρ* are calculated directly from the linear fit to eqn (3). Uncertainties in ℓ_c_ are taken from the respective standard deviations calculated from our set of measurements, whilst the uncertainty in *γ* is propagated from Δ*ρ* and ℓ_c_.

We apply the sessile drop technique to Ddx4^N^ model biomolecular condensates, formed by the liquid–liquid phase-separation of Ddx4^N^ 1-229 and Ddx4^N^ 1-231 in aqueous buffer. Fig. 2(a) shows exemplary Young-Laplace fits to sessile condensates of both Ddx4^N^ proteins, whose interfaces are clearly deformed away from spherical cap geometry by their mass. Sessile drop analysis was repeated for 13 Ddx4^N^ 1-229 and 11 Ddx4^N^ 1-231 samples, varying sample volume and protein concentration, concomitantly altering the sample density *ρ*_0_. In Fig. 2(b) we plot the ratio of condensate volume to sample volume *ν*_β_/*ν*_0_ against sample density, revealing a strong linear correlation, allowing measurement of Δ*ρ* and *ρ*_α_ by comparison to eqn (3). These data, well-described by constant values of *ρ*_α_ and Δ*ρ* as total protein concentration is varied (at constant temperature of 22 °C), support the modelling of our Ddx4^N^ condensates as a phase-separated mixture of protein and solvent – the basis for our subsequent Scheutjens–Fleer calculations. To further test the ability of eqn (3) to characterise Ddx4^N^ phase-separation, we directly measured protein concentration of the protein-poor phase *ρ*_α_ through spectrophotometry (see Section 6.4), yielding 27 ± 2 μM and 48 ± 2 μM for phase-separated Ddx4^N^ 1-229 and Ddx4^N^ 1-231 respectively. These values are in close proximity to those obtained from converting the expectation value of *ρ*_α_ into protein concentration, 32 μM and 54 μM for Ddx4^N^ 1-229 and Ddx4^N^ 1-231 condensates respectively. We note that we did not see any dependency of *ν*_β_/*ν*_0_ on sample volume *ν*_0_, which varied in the range 66–180 μL.

In Fig. 2(c) we plot the capillary lengths ℓ_c_ obtained from fitting eqn (2) to condensate profiles against sample density *ρ*_0_. Unlike that observed for *ν*_β_/*ν*_0_, we see no clear dependency of ℓ_c_ on *ρ*_0_, which, when combined with our observed linear relationships between *ν*_β_/*ν*_0_ and *ρ*_0_ in Fig. 2(b), supports a constant value of *γ* for each Ddx4^N^ condensate as total protein concentration is varied. We take the mean and standard deviation of ℓ_c_ of each Ddx4^N^ condensate to be good descriptors of their measured value and uncertainty respectively, yielding 293 ± 6 μM and 237 ± 5 μM for Ddx4^N^ 1-229 and Ddx4^N^ 1-231 respectively, permitting evaluation of condensate surface tensions using eqn (1) and our measured values of Δ*ρ*.

The numerical values obtained from sessile drop analysis are tabulated in Fig. 2(d). Our values for Ddx4^N^ condensate surface tensions, 82 ± 10 μN m^−1^ and 38 ± 3 μN m^−1^ are comparable to those measured for other model IDP condensate systems; PGL-3 (19.4 μN m^−1^),^9^ FUS_267_ (90 μN m^−1^)^27^ and LAF-1 RGG-RGG (159 μN m^−1^).^22^ Ijavi *et al.*^27^ also report a density difference of 135 kg m^−3^ for FUS_267_, of similar magnitude to our Ddx4^N^ condensates. Varying buffer conditions reported for published measurements hinder further comparisons, such as relating differences in condensate surface tension to differences in IDP sequence.

Our measurements reveal a decrease in absolute net charge (and a slight decrease in length) on Ddx4^N^ by one unit, from −2.1*e* for Ddx4^N^ 1-231 to −1.1*e* for Ddx4^N^ 1-229, roughly doubles their condensate's surface tension *γ* from 38 ± 3 μN m^−1^ to 82 ± 10 μN m^−1^ and increases the density difference Δ*ρ* by factor 1.4 from 69 ± 5 kg m^−3^ to 97 ± 10 kg m^−3^. The concentration of protein inside Ddx4^N^ condensates, extracted from density calculations, are 11.3 ± 0.8 mM for Ddx4^N^ 1-231 and 15.9 ± 1.8 mM for Ddx4^N^ 1-229. As Δ*ρ* quantifies the degree of phase-separation, these measurements are in agreement with our previous finding, where we showed a decreasing absolute protein charge stabilises Ddx4^N^ condensates,^19^ showing here that the same change destabilises their surface.

**Fig. 3:**
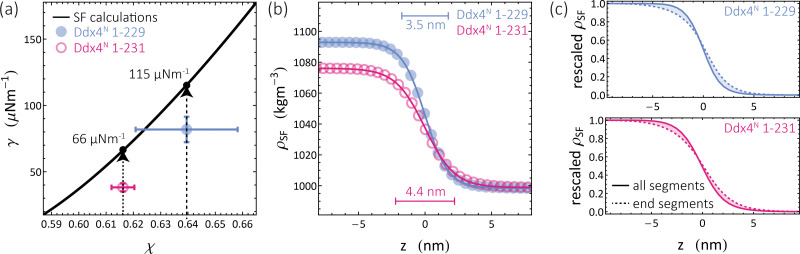
(a) Measured values of Ddx4^N^ condensate surface tensions *γ* and interaction parameters *χ* overlaid on the theoretical relationship (SF calculations) between *γ* and *χ* for homopolymers of length 234 and 236, calculated as detailed in Section 6.8. Note that the two curves exhibit only very minor differences (<1%) over the (*χ*,*γ*) range displayed, not visually resolvable on the plotted range. Dashed arrows emphasise the relationship between the interaciton parameter *χ*, measured values (coloured circles), and calculated values (black disks). (b) Calculated interfacial profiles for Ddx4^N^ 1-229 (blue) and Ddx4^N^ 1-231 (magenta), corresponding to the respective black points in (a). Solid lines indicate the fits to a hyperbolic tangent function (see text). (c) Scheutjens–Fleer interfacial profiles normalised to 1 in the protein-rich phase and 0 in the protein-poor phase for Ddx4^N^ 1-229 and Ddx4^N^ 1-231 condensates showing the distribution of all polymer segments and polymer end-segments.

Through eqn (9), we are able to define Flory–Huggins interaction parameters for each Ddx4^N^ condensate, allowing comparison with theoretical values of surface tension, calculated using Scheutjens–Fleer theory. Our experimental values of surface tension are plotted against the interaction parameter *χ* in Fig. 3(a) which also displays the theoretical dependency of *γ* on *χ* for homopolymers with length 234 and 236. Note that the difference in the two curves is very slight, and in Fig. 3(a) the two curves essentially overlay. Scheutjens–Fleer theory modestly overestimates the surface tension of both condensates, by a factor of 1.4 for Ddx4^N^ 1-229 condensates and a factor of 1.7 for the Ddx4^N^ 1-231 condensates.

The Scheutjens–Fleer theoretical compositional equilibrium profiles normal to the surface of each Ddx4^N^ condensate, corresponding to the two black points in Fig. 3(a), are plotted as densities in Fig. 3(b). The length scale of the compositional inhomogenity associated with these interfaces may be quantified by fitting^53^10
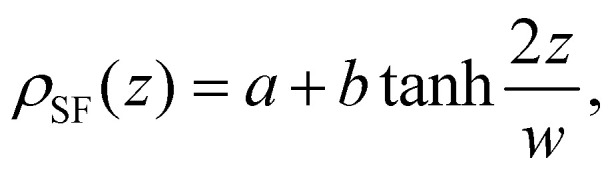
where *w* is the interfacial width and *a* and *b* are fitting parameters. Doing so reveals interfacial widths of 3.5 nm and 4.4 nm for the Ddx4^N^ 1-229 and 1-231 condensates respectively, similar to the reported hydrodynamic radii of another Ddx4^N^ variant differing by only a few amino acids (*R*_H_ = 3 nm).^18^ Given the <1% difference in length between Ddx4^N^ 1-229 and Ddx4^N^ 1-231, we attribute the sharper interface for Ddx4^N^ 1-229 condensate to its increased *χ* relative to Ddx4^N^ 1-231. In SI Fig. 5(a), the relationship between interaction parameter and interfacial width predicted by Scheutjens–Fleer theory is plotted for phase-separated binary solutions of homopolymers with varying length. The interfacial width decreases with both increasing interaction parameter and polymer length, which in turn, are parameters stabilising phase-separation. In SI Fig. 5(b) we see Scheutjens–Fleer theory predicts a decreasing interfacial width with decreasing surface tension, with only a weak dependency on polymer chain length.

We next used Scheutjens–Fleer calculations to investigate protein conformations at the interface, by performing calculations of interfacial profiles using homopolymers with labelled end-segments, corresponding to the N and C termini of Ddx4^N^ chains. Fig. 3(c) shows interfacial compositional profiles for all segements and end-segments, linearly rescaled to the range [0,1]. For both theoretical Ddx4^N^ condensate interfaces, there is a relative migration of end-segments towards the protein-poor phase, indicative of a conformational bias for proteins in the interfacial region to be arranged with their tail-ends pointing out of the condensate.

## 4 Discussion

Surface tension is a key thermodynamic parameter governing nucleation, coalescence and wetting behaviour of intracellular liquid-like biomolecular condensates.^2^ Recent research has focused on developing practical and computational^54,55^ methods to characterise the fluid–fluid interfaces in model biomolecular condensate systems, comprising aqueous phase-separated solutions of IDPs.^10,22–25,27^ Seeking to extend the toolkit for studying biomolecular condensate surface tensions, we have presented a straightforward experimental method for their measurement and investigated the applicability of Scheutjens–Fleer theory for their computation.

Combining widely accessible optical microscopy with standard measurements of protein concentration through spectrophotometry, we evaluated the capillary lengths, densities and surface tensions of Ddx4 model condensates. Previous measurements of model condensate surface tension, through characterisation of condensate coalescence events, droplet deformation under gravity and micropipette aspiration,^22,23,27^ rely on hydryodynamic modelling of condensate viscosities and sedimentation velocities. Measurements of such properties are complex for biomolecular condensate systems which exhibit ageing on the scale of hours,^56,57^ complicating the characterisation of their surface tension. Our method bypasses this issue, using only static properties of condensates to determine their surface tension and density, thus is applicable to a broader range of model biomolecular condensates than previously reported techniques.

Application of our experimental workflow to condensates formed by two Ddx4 proteins, differing by two amino acids and a unit charge, revealed surface tensions similar in magnitude to reported values for condensates formed by FUS_267_, PGL-3 and LAF-1-RGG-RGG IDPs, roughly characterised by the range 10–100 μN m^−1^.^9,22,27^ This energy scale is bordered either side by interfaces in colloid-polymer mixtures (*γ* ∼ 1 μN m^−1^) and complex coacervates (*γ* ∼ 100 μN m^−1^),^28,32^ and similar to recent surface tension measurements for native intracellular condensates (0.1–10 μN m^−1^).^58^ Condensates formed by Ddx4^N^ 1-231, with a net charge of −2.1*e* at pH 8.0, showed a decreased stability and surface tension, by factors of 1.4 and 2.1 respectively, relative to Ddx4^N^ 1-229 (net charge −1.1*e*). We and others,^19,59^ have previously rationalised the detrimental impact of increased absolute protein charge on condensate stability as a weakening of the mean-field interactions between proteins. The increased sensitivity, relative to condensate stability, of surface tension to the strength of intermolecular interactions, is in line with critical scaling theory for phase-separated fluids.^51,60^ This suggests our observed relationship between stability, surface tension and intermolecular interactions is a feature inherent in the phase-separated nature of Ddx4^N^ condensates.

Next we performed Scheutjens–Fleer calculations, obtaining theoretical values for surface tension and condensate densities. Interactions between polymer and solvent within our Scheutjens–Fleer calculations were parameterized by Flory–Huggins interaction parameters, obtained from our experimental measurements of Ddx4^N^ condensate densities. Theoretical surface tensions are within a factor of two of our measured values, overestimating surface tension in both cases. This semi-quantitative accuracy of Scheutjens–Fleer theory in calculating Ddx4^N^ condensate surface tensions suggests their interfacial phenomena, like their bulk phase behaviour, display the hallmarks of a phase-separated homopolymer–solvent mixture. This is likely a result of the weak interactions between constituent amino acids of IDPs,^61,62^ yielding IDP configurations dominated by chain entropy, leaning towards the random-mixing approximation inherent in mean-field theories.

Theoretical interfacial widths for Ddx4^N^ condensates on the order of nm, obtained from Scheutjens–Fleer density profiles normal to the interface, showed a slightly sharper interface for the more stable Ddx4 1-229 condensate. Consistent with our results, atomistic simulations of the phase-separation of IDPs LAF-1 RGG and FUS LC, similar in size and sequence composition to Ddx4^N^, by Zheng *et al.*,^55^ show interfacial widths similar to those calculated for Ddx4^N^, on the order of a few nm. Scheutjens–Fleer theory and polymer scaling laws^51^ predict increased surface tension, and decreasing interfacial width, in solutions of phase-separated polymers upon strengthening polymer–polymer interactions and increasing chain length. In the context of biomolecular condensates, this suggests nature may modulate their surface tension by either altering the amino acid composition of constituent IDPs to modify the strength of protein–protein interactions, or altering the length of disordered regions.

Finer investigation of the predicted interfacial structure, presented in Fig. 3(b), revealed a relative bias of polymer end-segments towards the protein-poor phase. The Scheutjens–Fleer framework computes molecular conformations using a weighted walk,^34^ with weights determined by the local mean molecular field. Placing inner-segments in the polymer-poor region costs relatively more free energy than end-segments, as they necessarily have two neighbouring segments, also located in the vicinity of an unfavourable region. Therefore end-segments are more likely to extend towards the unfavourable polymer-poor phase. This behaviour is similar to results obtained for the concentration profiles of polymer solutions in the vicinity of hard walls,^49,63^ where end-segments exhibit closer approach to the region devoid of polymer directly adjacent to the wall. Translated to biomolecular condensates formed by IDPs, this effect, driven by chain connectivity, should drive the exposure of terminal amino acids to the surface of condensates. Further investigation of these phenomena are crucial for understanding biochemical process at condensate interfaces, such as adsorption of RNA or interface-driven redox reactions.^64,65^

Of current interest are the biophysical determinants of the interfacial structure in multiphase condensates such as the nucleolus, stress granules and P-bodies.^2^ Modelling of such surface tension driven biomolecular condensate phenomena within the Scheutjens–Fleer framework is possible, provided relevant interaction parameters are known, and our results demonstrating its applicability to the interface in solutions of phase-separated Ddx4^N^, suggest it may be able to predict relevant surface tensions with a high degree accuracy. Equivalently, Scheutjens–Fleer calculations may serve as a reference point, giving the mean-field polymeric contribution to condensate interfaces, facilitating the development of further physical theories relating molecular interactions to biomolecular condensate interfacial phenomena. We hope the framework presented here will help further the distillation of the essential physical principles applicable to biomolecular condensate interfaces.

## 5 Conclusions

Our work demonstrates both a simple sessile drop technique to measure the surface tension and densities of Ddx4^N^ model biomolecular condensates, and the calculation of condensate surface tensions from condensate density, through mean-field Scheutjens–Fleer lattice computations. Reducing the charge of Ddx4^N^ by one unit decreased its phase-separation and interfacial tension similar to qualitative predictions from scaling arguments for fluid–fluid interfaces. The surface tensions of Ddx4^N^ condensates were reproduced by Scheutjens–Fleer theory, taking their measured mean-field interaction parameters as input, with near quantitative accuracy. Our results build on previous work demonstrating the applicability of Flory–Huggins models to the phase-separation of condensate-associated IDPs,^16,18,29,30^ showing the same sets of modelling assumptions may be used at their surfaces. These results demonstrate the utility of mean-field polymer theory in modelling IDP condensate interfaces, expanding our understanding of the key driving forces of their surface tension, and of fundamental aspects of intracellular organisation. Future work may focus on direct experimental interrogation of the interfacial structure at the surfaces of biomolecular condensate interfaces, allowing verification of the computational predictions presented here for Ddx4^N^ and elsewhere for other IDP condensates.^55^

## 6 Materials & methods

### 6.1 PEGylation of cuvettes

Surface passivation of the inner surface of cuvettes with PEG gave Ddx4^N^ condensate contact angles approaching 180°, maximising the vertical extension of sessile condensates, in the same manner as reported by Ijavi *et al.*^27^ for Bik1 and FUS_267_ condensates. Here, we adapt their PEGylation protocol for coating of quartz glass cuvettes (Hellma Semi-Micro Cuvettes AS4C-QS/QG, 10 mm path length). Quartz glass cuvettes were treated with oxgyen plasma for 10 min to remove any surface adsorbed organic contaminants. From a 10 mL toluene (99.8%, anhydrous from ThermoFisher), 16 μL HCL (fuming 37%, ACS reagent grade), 50 μL PEG silane ((3-[methoxy(polyethyleneoxy)propyl]trimethoxysilane, 6-9 PEG-units) bought from ABCR GmbH) solution, 1.5 mL was added to each cuvette. Cuvettes were sealed with their lids and scotch tape and left for 21 h in a fume hood. The PEGylation reaction solution was then decanted and cuvettes were washed with toluene and twice with ethanol before drying with clean air. PEGylated cuvettes were stored under vacuum until use.

### 6.2 Protein sequences

**Fig. 4:**
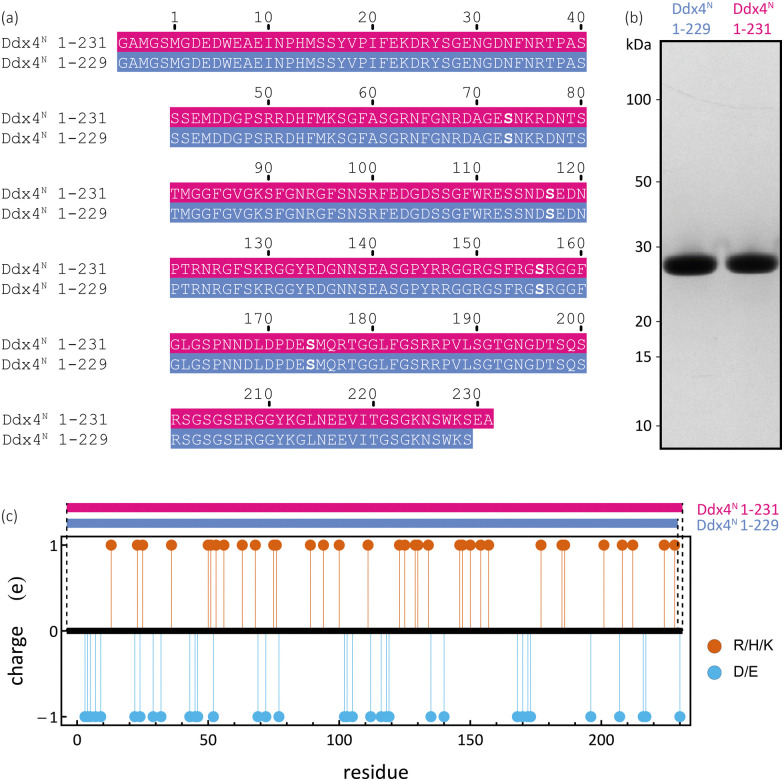
(a) Sequence alignment of the two Ddx4^N^ proteins used in this work, generated using JalView.^70^ Number labels correspond to the consensus Ddx4 sequence, preceeded here by a short GAMGS sequence, an artefact of restriction cloning used to prepare DNA plasmids for protein expression (see text). Positions of the cysteine to serine mutation, relative to the consensus Ddx4 sequence, are highlighted in bold font. (b) SDS-NuPage gel of purified Ddx4^N^ 1-229 and Ddx4^N^ 1-231 in adjacent lanes (see text). (c) Predicted charge per residue at pH 8 along the sequence of the Ddx4^N^ sequence studied in this work. The terminal residues of Ddx4^N^ 1-229 and Ddx4^N^ 1-231 are illustrated by dashed lines.

The sequence of the Ddx4^N^ proteins used in this work is given in Fig. 4(a). Ddx4^N^ 1-231 contains the first 231 residues of the consensus Ddx4 sequence, preceeded by GAMGS – an artefact of recombinant expression, with the four native cysteine residues swapped for serine (C73S, C117S, C156S, C174S), alleviating any propensity for disulfide bond formation. Ddx4^N^ 1-229 is equivalent to Ddx4^N^ 1-231 truncated at its C-terminus by two residues, equivalent to a 0.85% difference in length. The net charge on the proteins was calculated using the Henderson–Hasselbalch equation at pH 8 with the following p*K*_a_ values; C-terminus = 3.6, ASP = 4, GLU = 4.5, HIS = 6.4, N-terminus = 7.8, CYS = 8.14, TYR = 9.6, LYS = 10.4, ARG = 12.5.

### 6.3 Protein expression and purification

BL21(DE3) *E. coli* cells were transformed with IPTG inducible pET-SUMO 2HTb plasmids encoding the Ddx4^N^ sequence (Ddx4^N^ 1-229 or Ddx4^N^ 1-231), and grown in autoinduction media^66^ at 37C until reaching an optical density of 2. The temperature was then reduced to 18C and cells were left to express the Ddx4^N^ construct overnight. Cell pellets were resuspended in buffer (6 M guanidinium chloride, 10 mM imidazole, 20 mM sodium phosphate pH 7.4) and lysed by sonication. The His-tagged protein was purified by affinity chromatography using Nickel NTA Agarose Resin (Agarose Bead Technologies). ULP1 was then added to remove the tag, and the eluent containing the target protein was further purified through size exclusion chromatography, simultaneously exchanging the protein product into storage buffer (300 mM NaCl, 20 mM Tris pH 8). Ddx4^N^ was then concentrated to a concentration stable with respect to phase-separation at room temperature, but unstable with respect to phase-separation when diluted 1 : 1 with 20 mM Tris pH 8 at room temperature (99 μM for Ddx4^N^ 1-229 and 160 μM for Ddx4^N^ 1-231). Purified protein was then syringe filtered through 0.2 μM filters before snap freezing in liquid nitrogen and stored at −80 °C.

### 6.4 Protein characterisation

The purity of purified Ddx4^N^ was assessed using sodium dodecyl sulfate polyacrylamide gel electrophoresis (SDS-PAGE) in 12-well precast Invitrogen^TM^ NuPAGE^TM^ 4–12%, Bis–Tris polyacylamide gels (see Fig. 4(b)). Samples containing 3 μg protein were prepared at a ratio 3 : 1 with Invitrogen^TM^ NuPAGE^TM^ LDS Sample Buffer (4X), before boiling at 95C for 1 min. 10 μL of samples were then loaded onto gels, along with 8 μL PageRuler^TM^ Unstained Protein Ladder, serving as a standard, before running the gel at 200 V for 35 min. Gels were stained overnight with InstantBlue Coomassie.

The concentration of protein samples were measured using a NanoDrop^TM^ Lite Spectrophotometer (Thermo Fisher Scientific^TM^) by loading 1 μL sample and recording the absorbance of at 280 nm, relative to a baseline recorded for buffer solution containing no protein. The Beer–Lambert law^67^ then relates the absorbance to protein concentration through the molar decadic extinction coefficient; 23 950 M cm^−1^ for both Ddx4^N^ proteins used in this work. All concentration measurements were performed at least in triplicate. The absorbance measurements used in this work fall in the range 0.309–3.924, well within the detection limits of the spectrophotometer (0.08–30.00).

For measurements of the protein concentration in the protein-poor phase, 100 μL purified protein was heated to 40C and combined with 100 μL 20 mM Tris pH 8 in a 1.5 mL Eppendorf tube and allowed to cool to room temperature for 20 min, before centrifugation at 100 g for 10 min. The phase-separated solution was then held at 22 °C on a heating block for 15 min before 20 μL was aspirated and combined with 20 μL 300 mM NaCl 20 mM Tris pH 8. The protein concentration in this solution, measured through spectrophotometry, was taken to be half that of the protein concentration in the protein-poor phase.

### 6.5 Density calculations

To determine the densities *ρ*_0_ of our samples, we utilised the following formula11
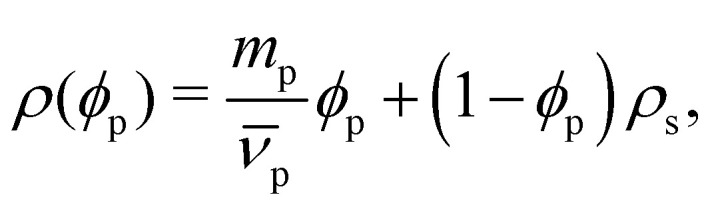
where *m*_p_ is the molar mass of the respective protein (25 109.54 g mol^−1^ and 25 309.74 g mol^−1^ for Ddx4^N^ 1-229 and Ddx4^N^ 1-231 respectively), *ν̄*_p_ is the molar volume of the protein calculated using tabulated values provided by Zhao;^68^ 19020.3 cm^3^ mol^−1^ and 19169.7 cm^3^ mol^−1^ for Ddx4^N^ 1-229 and 1-231 respectively. We determined the solvent density *ρ*_s_ by weighing incremental volumes of 150 mM NaCl 20 mM Tris pH 8.0, returning *ρ*_s_ = 998.2 kg m^−3^. The protein volume fraction *ϕ*_p_ is calculated from protein concentration *c*_p_ through

12 *ϕ*_p_ = *c*_p_*ν̄*_p_.

Combining eqn (11) and (12) allows inter-conversion of densities, volume fractions and protein concentrations.

### 6.6 Sample preparation and imaging

Recombinantly expressed and purified Ddx4^N^ in 300 mM NaCl, 20 mM Tris pH 8 was removed from cryogenic storage and allowed to heat ambiently to room temperature. Purified protein was then diluted to a concentration double that desired for phase-separation experiments. Both purified protein and a solution of 20 mM Tris pH 8 were heated to 40C on a heating block and then combined 1 : 1 in a 1.5 mL Eppendorf tube. 66–180 μL of this solution was then added to a PEGylated quartz glass cuvette prepared as detailed above. Ambient cooling of the sample to 22 °C initiates liquid–liquid phase-separation, forming microscopic Ddx4^N^ condensates. 10 min after visible phase-separation, cuvettes were centrifuged at 20 g in a benchtop centrifuge for 10 min, set to 22 °C, driving the coalescence of microscopic condensates into a single, large (∼300 μm in characteristic dimension), Ddx4^N^ condensate. A control experiment, where condensate coalescence was induced by leaving a tilted sample on the benchtop overnight, showed the capillary length of our condensates to be unaffected by centrifugation.

Axial symmetry of sessile condensates, required for application of eqn (2) and (3), was checked through naked eye inspection before imaging, see Fig. 1(a) for an image of a phase-separated sample of Ddx4^N^ 1-231 following centrifugation captured using a phone camera. We characterised the evaporation rate of our sample setup as 10 μL every 24 h, by recording the mass of three samples over three days. To minimize the impact of volume changes due to evaporation, we imaged samples within 3 h of preparation. Sessile condensates were imaged using a Olympus BX43 microscope rotated by 90° relative to the vertical. Before imaging, the inclination of both the microscope and stage were checked to be horizontal using a spirit level, and the ambient temperature was checked to be 22 °C using a red spirit filled thermometer (Brannan). Grayscale 2560 × 1920 bright-field images of sessile condensates were captured using an Olympus SC50 camera with an Olympus PLN 4× Objective, see Fig. 1(b) for an image of our microscopy setup. In the case of a sample containing multiple sessile condensates, centrifugation was repeated to drive their coalescence.

### 6.7 Image analysis

Mathematica 13^69^ was used to extract and fit profile coordinates from grayscale images of sessile Ddx4^N^ condensates (see Fig. 1). The image coordinates describing the fluid–fluid surface of sessile condensates were extracted using the EdgeDetect function, combining the left and right-hand side of the interface to give a single profile. Eqn (2) is fit to (*r*,*h*) coordinates by integrating over *s* from 0 to *s*_max_, where the latter is defined as 1.8× the total Euclidean distance between adjacent (*r*,*h*) coordinates, with a MeanFilter of radius 5 applied to the set of *r* values. This integration generates a test profile for input values of *R*_0_ and ℓ_c_, which are optimised using the NMinimize function, where the best fit values minimize the total Euclidean distance between profile coordinates and the generated profile. To obtain condensate volumes, we recalculate *s*_max_ as the first value of *s* which reproduces the measured height of condensates. The condensate volume is then obtained from the optimum *ψ*(*s*) profile (given by integrating eqn (2)) by integrating13
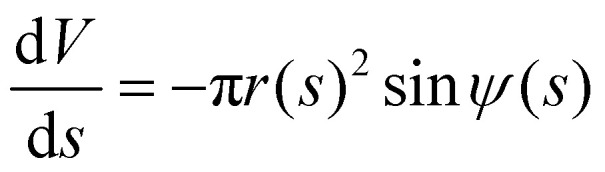
from 0 to *s*_max_, where *r*(*s*) is defined by14
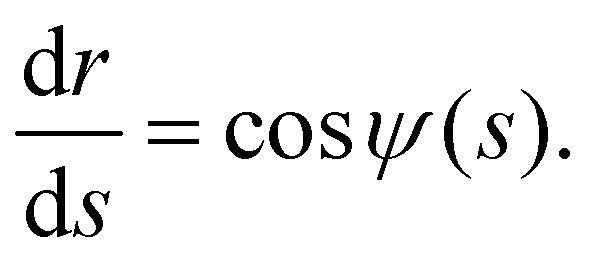
Condensate volumes were in the range 50–300 nL.

### 6.8 Scheutjens–Fleer calculations

The Scheujens–Fleer Self-Consistent Field (SF-SCF) computations are performed by solving the SF-SCF mean-field equations numerically on a lattice. We follow the procedure of Scheutjens and Fleer.^33^ SF-SCF basically can simulate (co-)polymeric, colloidal and/or surfactant systems, usually in a solvent, in a mean-field fashion. In general, SF-SCF computations can be performed after defining the geometry, the components present and the direct interactions between all components in the system through Flory–Huggins parameters. Electrostatic interactions can additionally be accounted for at the Gouy–Chapman level. Rather than explaining the details, we refer the interested reader to excellent reviews on the method,^34,37^ and describe herein the relevant input parameters used in this work. All forces in the SF-SCF computations are the result of direct interactions between all components quantified through Flory–Huggins parameters. Each chemical entity in the SF-SCF computations is considered as taking up a single lattice unit. We performed the computations using the widely spread SFbox software developed by F. A. M. Leermakers. Recently, the source code, called namics, has been made available: https://github.com/leermakers/Namics/. Running SFbox on a laptop computer returned optimum compositional profiles and interfacial tension in a matter of seconds.

Within the input file for SFbox, we constructed Ddx4^N^ 1-229 and Ddx4^N^ 1-231 effective homopolymers as molecules comprising 234 and 236 monomers respectively (see Fig. 4). Calculations were performed on a 200 layer 3-D lattice with a gradient in one direction and mirrored boundary conditions. The lattice was populated with 12 polymer molecules, giving a volume fraction of 0.06 – close to the critical volume fraction of 0.061 (to 2 s.f.) predicted by Flory–Huggins theory for both chains. For initial calculations, the interaction parameter between polymer and solvent sites was initially set to 0.741 658 and polymers were restricted to the first 30 lattice sites. In subsequent calculations previous output compositional profiles were used as initial configurations, polymers segments were unrestricted and the interaction parameter was decreased in units of 0.005 until *χ* decreased below its critical value. In this series, the exact values of *χ* recorded from our experiments (see Fig. 2(a)) were also present: 0.641 for Ddx4^N^ 1-229 and 0.616 for Ddx4^N^ 1-231 (both to 3 s.f.). This technique ensured the generation of only one interface in our calculations. The surface tensions of each profile were calculated using eqn (8). Interpolation was performed (using the Interpolation function in Mathematica 13^69^) allowing continuous evaluation of *γ* as a function of *χ*, yielding the plots in Fig. 3(a). Test calculations performed with larger lattices, keeping the total volume fraction of polymer constant, showed minimal uncorrelated changes in surface tension. Scheutjens–Fleer lattice profiles shown in Fig. 3 are transformed from simulation units to real space using the lattice size *b* obtained from the mean volume per amino acid calculated using standard values as detailed in Sections 2.1 and 2.2, amounting to 5.12 Å (to 3 s.f.) for each protein.

**Fig. 5:**
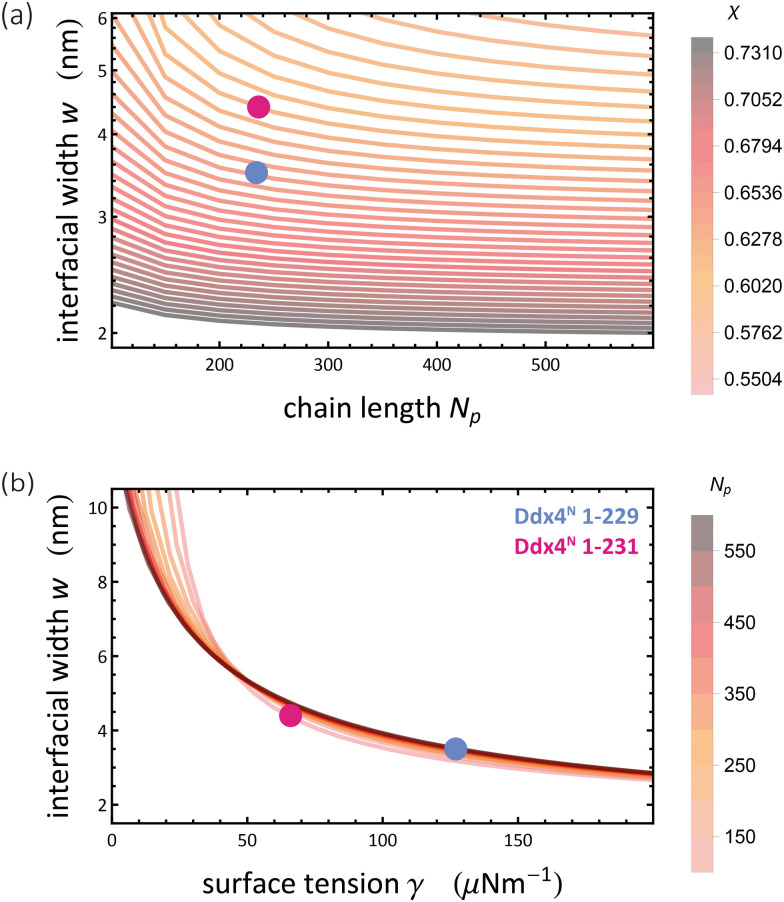
Interfacial width was a function of (a) chain length *N*_p_ and interaction parameter *χ* and (b) surface tension *γ* and chain length *N*_p_, obtained from Scheutjens–Fleer calculations with *λ* = 1/6 and *b* = 5.12 Å, representing a cubic lattice populated with Ddx4^N^-like chains. Widths were calculated by fitting eqn (10) to volume fraction profiles. Coloured blobs indicate our calculated values for Ddx4^N^ 1-229 and Ddx4^N^ 1-231 condensates.

The profiles presenting end-segment distributions shown in Fig. 3(b) were generated from SFbox calculations using the same technique as outlined above, except the terminal monomers comprised a third species, permitting the extraction of their compositional profile. Data in Fig. 5 were generated using the same calculation procedure detailed above, sweeping over increasing *χ* for each polymer length *r*.

## 7 Supplementary information

### 7.1 Curvature effects on phase equilibria

In a binary mixture of solvent and protein, the impact of interface curvature on the binodal may be accounted for by considering the Laplace pressure. For a two-phase system with a flat interface, the equilibrium constraint on the protein volume fraction is

15 *μ*_p,α_(*T*,*ϕ*_p,α_,*P*) = *μ*_p,β_(*T*,*ϕ*_p,β_,*P*).

The curvature of a droplet of phase β immersed in phase α with radius *R* increases the internal pressure of the droplet by^52^16
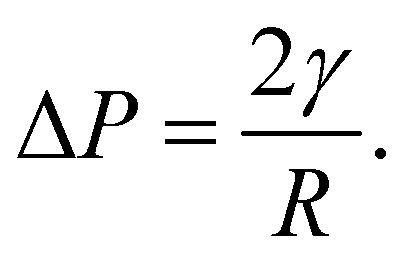
This Laplace pressure augments the chemical potential of the protein in phase β by a factor *ν̄*_p_Δ*P*, where *ν̄*_p_ is the molar volume of the protein, yielding17
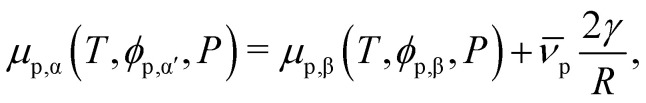
where *ϕ*_α′_ denotes the new equilibrium volume fraction of protein in phase α. To illustrate the impact of Laplace pressure on the two-phase equilibria present in our Ddx4^N^ system, we consider a droplet with a radius of *R* = 200 μm and surface tension of 100 μN m^−1^. Its Laplace pressure, given by eqn (16), is equal to 1 Pa, which for a condensate formed by a Ddx4^N^ chain comprising 234 monomer units, with interaction parameter *χ* = 0.641 and molecular volume ν̄_p_ = 31.6 nm^3^, results in an expected increase of *ϕ*_p,α_ by less than 10^−3^% (from 6.14486 × 10^−4^ to 6.14491 × 10^−4^).

## Author contributions

Jack Holland: conceptualization, methodology, formal analysis, investigation, writing – original draft, writing – review & editing. Alfonso A. Castrejón-Pita: methodology, resources, writing – review & editing. Remco Tuinier: software, writing – review & editing. Dirk G. A. L. Aarts: conceptualization, methodology, writing – review & editing, supervision, funding acquisition. Timothy J. Nott: conceptualization, methodology, resources, writing – review & editing, supervision, project administration, funding acquisition.

## Conflicts of interest

There are no conflicts to declare.

## Supplementary Material
